# A New Surgical Technique of Pancreaticoduodenectomy with Splenic Artery Resection for Ductal Adenocarcinoma of the Pancreatic Head and/or Body Invading Splenic Artery: Impact of the Balance between Surgical Radicality and QOL to Avoid Total Pancreatectomy

**DOI:** 10.1155/2014/219038

**Published:** 2014-06-12

**Authors:** Ryosuke Desaki, Shugo Mizuno, Akihiro Tanemura, Masashi Kishiwada, Yasuhiro Murata, Yoshinori Azumi, Naohisa Kuriyama, Masanobu Usui, Hiroyuki Sakurai, Masami Tabata, Shuji Isaji

**Affiliations:** Department of Hepatobiliary Pancreatic and Transplant Surgery, School of Medicine, Mie University, 2-174 Edobashi, Tsu, Mie 514-0001, Japan

## Abstract

For pancreatic ductal adenocarcinoma (PDAC) of the head and/or body invading the splenic artery (SA), we developed a new surgical technique of proximal subtotal pancreatectomy with splenic artery and vein resection, so-called pancreaticoduodenectomy with splenic artery resection (PD-SAR). We retrospectively reviewed a total of 84 patients with curative intent pancreaticoduodenectomy (PD) for PDAC of the head and/or body. These 84 patients were classified into the two groups: conventional PD (*n* = 66) and PD-SAR (*n* = 18). Most patients were treated by preoperative chemoradiotherapy (CRT). Postoperative MDCT clearly demonstrated enhancement of the remnant pancreas at 1 and 6 months in all patients examined. Overall survival rates were very similar between PD and PD-SAR (3-year OS: 23.7% versus 23.1%, *P* = 0.538), despite the fact that the tumor size and the percentages of UICC-T4 determined before treatment were higher in PD-SAR. Total daily insulin dose was significantly higher in PD-SAR than in PD at 1 month, while showing no significant differences between the two groups thereafter. PD-SAR with preoperative CRT seems to be promising surgical strategy for PDAC of head and/or body with invasion of the splenic artery, in regard to the balance between operative radicality and postoperative QOL.

## 1. Introduction

When pancreatic ductal adenocarcinoma (PDAC) of the head and/or body invades the origin of splenic artery (SA), we usually cannot be able to avoid total pancreatectomy (TP) because the blood supply of distal pancreas becomes scarce after dividing the origin of splenic artery. Prognosis of PDAC patients following TP, however, has not overcome that of pancreaticoduodenectomy (PD) [1–3]. Moreover, TP causes insulin dependent diabetes mellitus (DM) and exocrine insufficiency, leading to a poor quality of life (QOL). DM after TP means a complete lack of endogenous insulin and glucagon, leading to uncontrollable frequent and deep states of hypoglycemia with hyperglycemic episodes (brittle diabetes) [3]. Recently, favorable perioperative control of blood glucose levels for patients with TP has been reported by using an artificial endocrine pancreas during the perioperative term [4] or at an outpatient clinic by using continuous subcutaneous insulin infusion pumps [5]. Nevertheless, inevitable insulin therapy, presence of brittle DM, and malabsorption after TP lead to poor QOL. Therefore, if the surgical margin status could be a microscopically negative (R0), TP should be avoided.

For the tumors with invasion of the SA, we had developed a new surgical technique of proximal subtotal pancreatectomy with splenic artery and vein resection, so-called pancreaticoduodenectomy with splenic artery resection (PD-SAR), usually in consideration of the balance between operative radicality and postoperative QOL. Blood flow to the pancreas tail can be obtained by the left gastroepiploic artery (LGEA) and/or posterior epiploic artery (PEA) even if we have to resect the left gastric artery (LGA) combined with total gastrectomy and splenectomy [6]. Previously, proximal subtotal pancreatectomy was performed by preserving SA to maintain blood supply of the pancreatic tail [7, 8]. Our procedure of PD-SAR was inspired by Sutherland et al. [9] and Warshaw [10] technique for distal pancreatectomy with preservation of the spleen which resects the SA and vein along with the pancreas but with careful preservation of the vascular collaterals in the splenic hilum.

The aim of the present study was to evaluate the significance of PD-SAR by examining surgical outcomes, RPV, and prognosis in comparison with those of conventional PD, paying special attention to postoperative pancreatic functions, total daily insulin dose, and nutritional status using TP as a control.

## 2. Patients and Methods

We retrospectively reviewed a total of 84 patients who had consecutively undergone curative intent pancreaticoduodenectomy (PD) for PDAC of the head and/or body at the Mie University Hospital between January 2008 when we experienced the first case with PD-SAR and December 2013. These 84 patients were classified into the two groups: conventional PD (*n* = 66) and PD-SAR (*n* = 18). Most patients were treated by preoperative chemoradiotherapy (CRT): gemcitabine-based CRT (G-CRT) (40 Gy radiation in 25 fractions with weekly intravenous infusion of gemcitabine 800 mg/m2 for 5 weeks including one-week break) [11, 12] or gemcitabine plus S1-based CRT (GS-CRT) (50.4 Gy radiation in 28 fractions with biweekly intravenous infusion of gemcitabine 600 mg/m2 for 8 weeks and oral S-1, active combination of tegafur, gimeracil, and oteracil, 60 mg/m2/day from day 1 to day 21 and from day 29 to day 49). We compared the two groups with respect to prognosis, postoperative pancreatic functions, and nutritional status.

### 2.1. Indication and Surgical Procedure of PD-SAR

**Figure 1:**
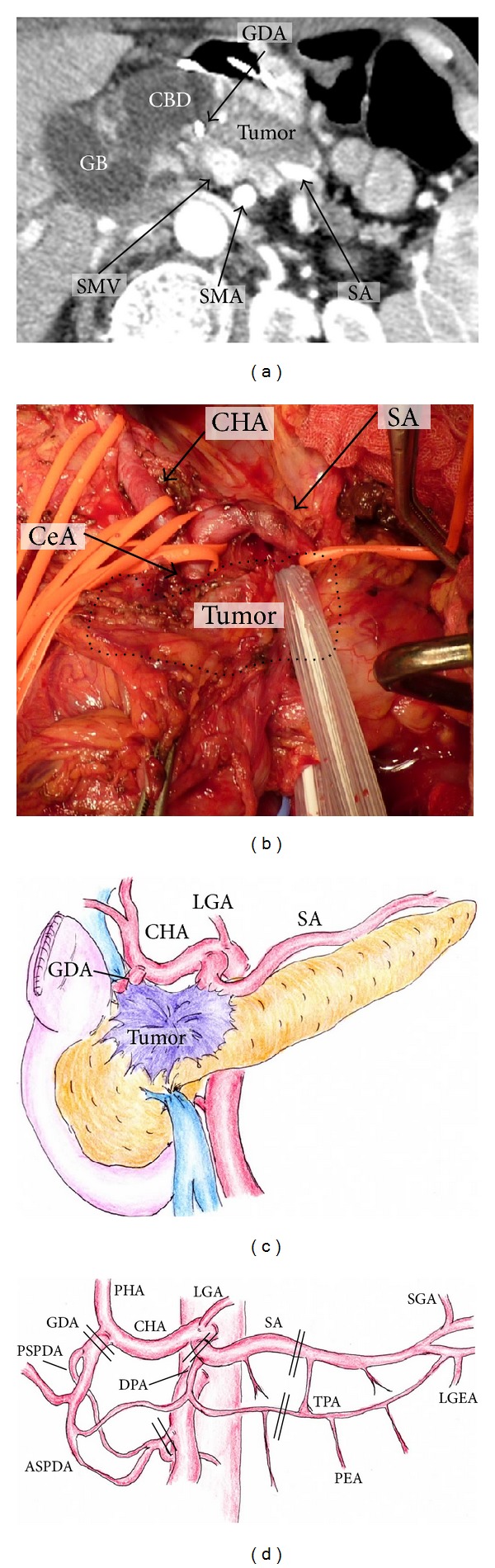
Indication of PD-SAR according to MDCT findings (a), intraoperative findings ((b) dotted circle indicates tumor border and schema of intraoperative findings) (c), and the arterial anatomy around the pancreas ((d) double line indicates cutting sites of artery). GB: gallbladder. CBD: common bile duct. SMV: superior mesenteric vein. SMA: superior mesenteric artery. SA: splenic artery. CeA: celiac artery. PHA: proper hepatic artery. LGA: left gastric artery. CHA: common hepatic artery. GDA: gastroduodenal artery. PSPDA: posterior superior pancreaticoduodenal artery. ASPDA: anterior superior pancreaticoduodenal artery. DPA: dorsal pancreatic artery. TPA: transverse pancreatic artery. SGA: short gastric artery. LGEA: left gastroepiploic artery. PEA: posterior epiploic artery.

We determined the indication for PD-SAR for PDAC patients as follows: pancreatic head and/or body tumor invading the proximal site of SA as well as gastroduodenal artery (GDA) according to preoperative multidetector computed tomography (MDCT) and intraoperative findings (Figures 1(a)–1(c)). MDCT was performed according to a defined pancreas protocol as four-phasic contrast-enhanced MDCT with thin slices at intervals of 1 mm [12]. We usually determined the indication of PD-SAR according to initial MDCT findings. After CRT, tumor abutment of SA was almost unchanged on MDCT even when the tumor size decreased. Therefore, indication of PD-SAR did not change before and after CRT. However, one patient who was scheduled to perform PD-SAR underwent conventional PD, because SA could be easily dissected from the tumor.

Since 2005, in our institution, surgical procedures of PD for PDAC of the head had been standardized for resection technique as anterior approach to the superior mesenteric artery [13, 14] according to the concepts of radical antegrade modular pancreatosplenectomy by Strasberg et al. [15] and no-touch isolation technique by Hirota et al. [16], and for pancreaticojejunostomy as pair-watch suturing technique [17].

Surgical procedures of PD-SAR are similar to those of PD except for combined resection of SA and vein, and total gastrectomy and splenectomy if necessary. As shown in Figure 1(d) indicating arterial anatomy around the pancreas and cutting sites of artery, the blood supply of the remnant pancreas is provided by the short gastric arteries (SGA), LGEA, and PEA. At surgery, adequacy of blood supply of the pancreatic tale and spleen is confirmed by the presence of arterial bleeding from the cut surface of the remnant pancreas and by color change of the spleen. If the spleen color becomes dark, splenectomy is performed with carefully preserving LGEA. When the tumor additionally invades the LGA, we perform combined resection of LGA followed by total gastrectomy and splenectomy if curative-intent resection is possible. In such case, the blood supply of the remnant pancreas is provided by PEA alone.

**Figure 2:**
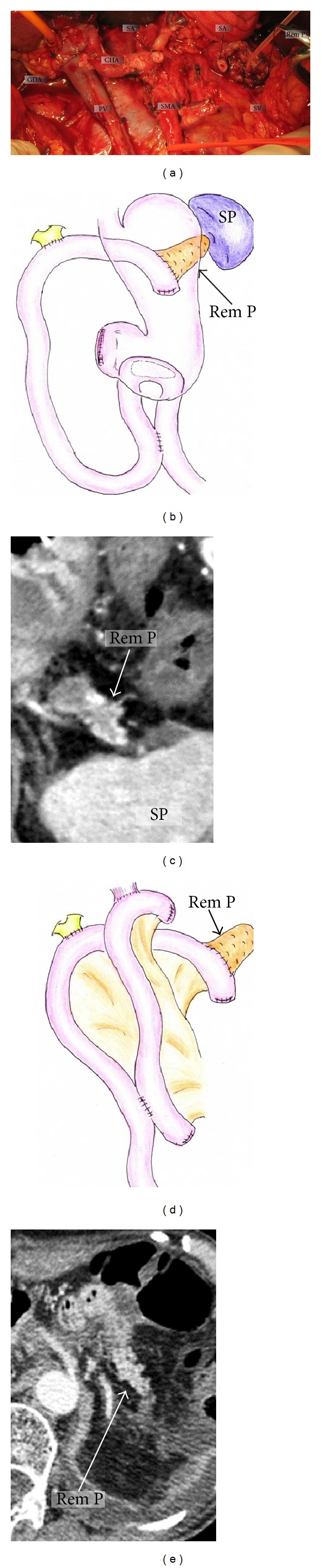
Intraoperative findings after PD-SAR (a) and schemas of reconstruction after PD-SAR and postoperative MDCT showing clear enhancement of the remnant pancreas (b)–(e). (b) and (c): schema of reconstruction after subtotal stomach preserving PD-SAR and postoperative MDCT. (d) and (e): schema of reconstruction after PD-SAR with total gastrectomy and splenectomy and postoperative MDCT. PV: portal vein. SMA: superior mesenteric artery. SA: splenic artery. CHA: common hepatic artery. GDA: gastroduodenal artery. SV: splenic vein. Rem P: remnant pancreatic parenchyma. SP: spleen.

Figure 2(a) shows intraoperative findings after PD-SAR. As of reconstruction procedures, end-to-side pancreaticojejunostomy is performed using the pair-watch suturing technique (PWST) [17], and hepaticojejunostomy is performed by interrupted or continuous suture, followed by gastrojejunostomy and Braun's anastomosis (Figure 2(b)). The blood supply of the remnant pancreas is clearly demonstrated on postoperative MDCT (Figure 2(c)).  Figure 2(d) shows the schema of reconstruction after PD-SAR with total gastrectomy and splenectomy, and Figure 2(e) clearly demonstrates enhancement of the remnant pancreas on postoperative MDCT. When the pancreatic duct is too small to perform duct-to-mucosa pancreaticojejunostomy because the remnant pancreas becomes very small, we perform dunking pancreaticojejunostomy.

**Figure 3:**
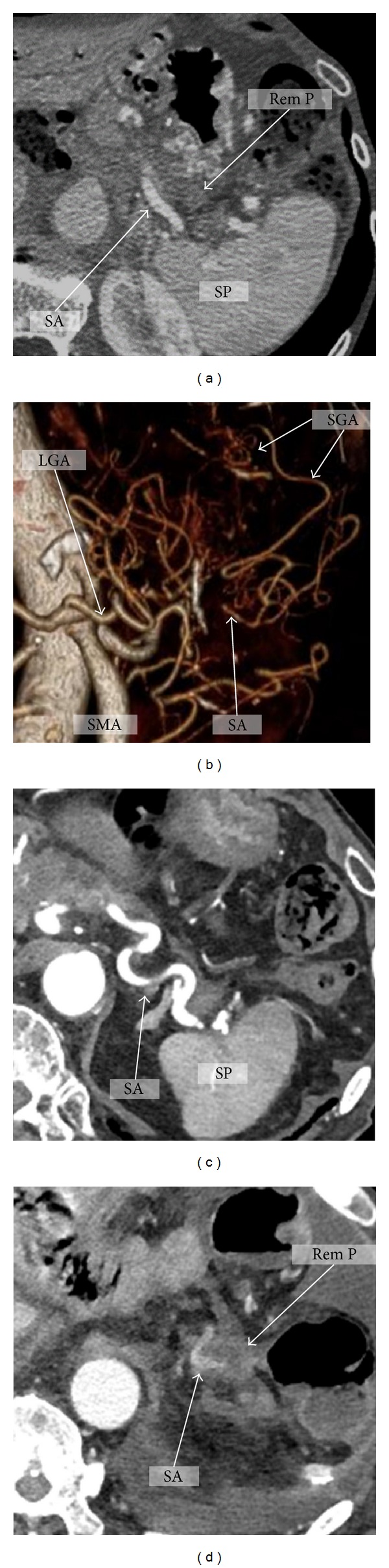
The arterial supply of the remnant pancreas after PD-SAR. MDCT (a) and 3D CT angiography (b) after subtotal stomach preserving PD-SAR showing SA clearly enhanced from SGAs anastomosing with LGA. Pre- (c) and postoperative MDCTs (d) in PD-SAR with total gastrectomy and splenectomy: SA and Rem P are enhanced even after PD-SAR with total gastrectomy and splenectomy, probably from PEA. LGA: left gastric artery. SGA: short gastric artery. SMA: superior mesenteric artery. SA: splenic artery. SP: spleen. Rem P: remnant pancreas. PEA: posterior epiploic artery.

The arterial supply of the remnant pancreas after PD-SAR is demonstrated in Figure 3. MDCT (Figure 3(a)) and 3D CT angiography (Figure 3(b)) after subtotal stomach preserving PD-SAR show that SA is clearly enhanced from SGAs anastomosing with LGA. Pre- (Figure 3(c)) and postoperative MDCTs (Figure 3(d)) in PD-SAR with total gastrectomy and splenectomy demonstrate that SA and the remnant pancreas are enhanced probably via PEA. As of feeding artery for PEA, our previous report demonstrated on postoperative angiography that the middle colic artery was the source of blood supply of PEA which fed SA [6].

### 2.2. Preoperative Characteristics, Surgical Outcomes, and Pathological Findings

We compared various factors in the patients between PD and PD-SAR, including (1) preoperative characteristics such as gender, age, size of tumor before treatment, International Union for Cancer Control (UICC)-T factor, resectability according to National Comprehensive Cancer Network guideline 2010 [18], cancer involvement of major vessels, and treatment before surgery and preoperative CA19-9 level, (2) surgical outcomes such as intraoperative blood loss, operation time, combined resection of major vessel or another organ, type of pancreaticojejunostomy anastomosis, intraoperative blood transfusion, degree of postoperative complications according to the Clavien-Dindo (C-D) classification [19], and duration of hospital stay (DHS), and (3) pathological findings of the resected specimen such as size of tumor, UICC-T factor, histological type, lymph node metastasis, degree of lymphatic invasion, venous invasion and intrapancreatic nerve invasion according to classification of pancreatic carcinoma of Japan Pancreatic Society [20], histological effect according to Evans' grading system for chemoradiation treatment effect [21], and surgical margin status (R0, R1, and R2).

### 2.3. Postoperative Chemotherapy and Follow-Up

From 6 weeks after operation, we made arrangement to start the adjuvant chemotherapy, consisting of gemcitabine at a dose of 800 mg/m2 biweekly or S1 60 mg/m2/day for 4 weeks followed by 2-week break for at least 6 months. All patients were evaluated as follows: physical examination every month; laboratory tests including CEA serum levels (normal < 5 ng/mL) and CA19-9 levels (normal < 37 U/mL) every 2 or 3 months; and MDCT every 3 months within 2 years, and thereafter every 6 months. All patients after PD, PD-SAR, and TP were given pancreatic enzyme, but the time of initiating and dosage of pancreatic enzyme supplementation were determined by each surgeon. The pancreatic enzyme supplementation was performed by pancreatin of 6 to 12 g/day or pancrelipase of 1800 or 3600 mg/day. The time of initiating and type of diabetes mellitus (DM) treatment were determined by each surgeon or DM specialist.

### 2.4. Measurement of the Remnant Pancreatic Volume

We measured the remnant pancreatic volume (RPV) by CT volumetry at 1 and 6 months after pancreatectomy. Serial transverse enhanced CT scan images were obtained at 1 and 1.25 mm interval. Each slice of the remnant pancreatic parenchyma was traced, and the corresponding area was calculated as the sum of pancreatic tissue area. Splenic vein and dilated pancreatic duct (3 mm or more) were excluded.

### 2.5. Prediction of Postoperative Pancreatic Functions Using Several Markers

Because exact methods for evaluation of pancreatic endocrine and exocrine functions are expensive and labor intensive, and, furthermore, insulino-acinar-ductal-incretin gut hormonal axis influences endo- and exocrine functions each other, which in turn makes it difficult to discriminate each other [22], there has been an increased need in clinical practice for a simple and widely available screening tool for detection of pancreatic functions. Lindkvist et al. [23] reported significance of nutritional markers such as albumin, prealbumin, magnesium, HbA1C, and cholesterol to predict the probability of pancreatic exocrine insufficiency. Furthermore, Yadav et al. [24] have recently suggested that decreased levels of serum amylase in type 2 DM are associated with decreased pancreatic function. To predict the remnant pancreatic functions in the present study, therefore, we examined type of DM treatment, total daily insulin dose, fasting blood sugar (FBS) level, HbA1c, serum amylase level, degree of body weight loss, serum albumin level, serum cholesterol level, and frequency of evacuation before and 1, 3, 6, and 12 months after pancreatectomy. In the present study, the patients were diagnosed as DM when either one of fasting blood sugar of 126 mg/dL or more or HbA1c of 6.5% or more was found or when DM treatment had been introduced preoperatively. As a control for PD and PD-SAR, we measured the same parameters in the 6 patients who underwent total pancreatectomy (TP) during the study period: PD-SAR was converted to TP in 2 and remaining 4 underwent resection of the remnant pancreatic head due to tumor occurrence (PDAC in 2 and intraductal papillary mucinous adenocarcinoma in 2) in the pancreatic head after distal pancreatectomy for PDAC in 1 and for intraductal papillary mucinous neoplasm in 3. The reason why the number of TP was very small as a control group was because we had been avoiding TP as much as possible by aggressively employing the procedure of PD-SAR.

### 2.6. Glucagon Stimulation Test

Because the blood supply of the remnant pancreas becomes scarce after PD-SAR, it is crucial to determine whether islets cells are functional or not. Oral glucose tolerance test (OGTT) provides a stimulus for the release of C-peptide from the islet cells which is equally as effective as intravenous glucagon injection test, that is, glucagon stimulation test (GST) [25]. OGTT after PD or PD-SAR is highly influenced by the types of gastrointestinal reconstruction, while GST is not. Therefore, GST was performed in the morning after an overnight fast: serum levels of C-peptide immunoreactivity (CPR) were measured in blood sample taken before (pre-CPR) and 10 minutes (post-CPR) after 1 mg of glucagon was intravenously injected. Δ CPR was calculated as (post-CPR-pre-CPR).

### 2.7. Statistical Analyses

All continuous values were presented as mean ± SD according to results of Fisher's distribution. Continuous variables were compared using Student's *t*-test, and categorical variables were compared using Pearson's chi-squared test.

In all patients, the date of the initial treatment was chosen as the starting point for the measurement of survival time. Recurrence-free survival time was defined as the time from the date of initial treatment to the date of first relapse or death. Overall and recurrence-free survival was calculated using the Kaplan-Meier method and was compared between the groups using the log rank test. The day of final follow-up was January 31, 2014, and there was no loss of follow-up. All statistical analyses were performed using SPSS version 21 (SPSS Inc., Chicago, IL) software. A *P* value < 0.05 was considered as being statistically significant.

## 3. Result

### 3.1. Preoperative Characteristics

The patients' background and preoperative clinical findings in the two groups are listed in Table 1. The mean size of tumor before treatment and the percentages of UICC-T4 and involvement of hepatic artery (HA), celiac artery (CeA), and splenic artery (SA) were markedly higher in PD-SAR than in PD, although the status of resectability according to NCCN guideline showed no difference between two groups. The rate of female was significantly higher in PD-SAR than in PD (*P* = 0.029), while there was no difference in the mean age of patients between two groups. Basically, our institutional policy to treat UICC-T3 and T4 PDAC patients, especially BR and UR, is to undergo CRT before surgery, as we previously reported [11, 12]. Among the total of 84 patients, we performed CRT before surgery in 75 patients (89.3%), chemotherapy alone in 4 (4.8%), and no treatment before surgery in 5 (5.9%). Among 18 patients with PD-SAR, 16 (88.9%) underwent preoperative CRT, and the remaining 2 who did not receive CRT had multiple (two) tumors in the head and body, of which body tumor invaded SA. Between the two groups, however, there were no differences in the type of preoperative treatment. Serum CA19-9 levels before and after preoperative treatment did not differ between the two groups.

### 3.2. Surgical Outcomes

Between PD and PD-SAR, there were no significant differences in surgical outcomes including blood loss, operation time, blood transfusion, degree of postoperative complications, and DHS, except for the rates of combined resection of SA and dunking pancreaticojejunostomy (Table 2).

### 3.3. Pathological Findings of Resected Specimen

As shown in Table 3, pathological tumor size was larger in PD-SAR than in PD, although there was no statistical difference in the two groups (*P* = 0.098). Pathological T classification did not differ between the two groups, although preoperative T classification was significantly different. As of histological effect of CRT, the incidence of grade IIb or more was higher in PD than in PD-SAR: 27/59 (45.8%) versus 3/16 (18.8%) (*P* = 0.083). The remaining factors such as UICC-stage, JPS-stage, histological type, lymph node metastasis, degrees of lymphatic, venous and intrapancreatic nerve invasions, and status of surgical margin showed no significant differences between the two groups. As of surgical margin, there were no patients with pancreatic cut margin positive in both groups, and the sites of R1 were unexceptionally dissected margins around SMA and/or HA and/or CeA in both groups. The causes of R2 in 2 cases with PD-SAR were macroscopic positive dissected margin around the common hepatic artery and solitary liver metastasis which was palliatively resected by partial hepatectomy, respectively. The cause of R2 in 1 case with PD was solitary liver metastasis which was palliatively resected by partial hepatectomy.

### 3.4. Overall Survival and Recurrence-Free Survival Rates

**Figure 4:**
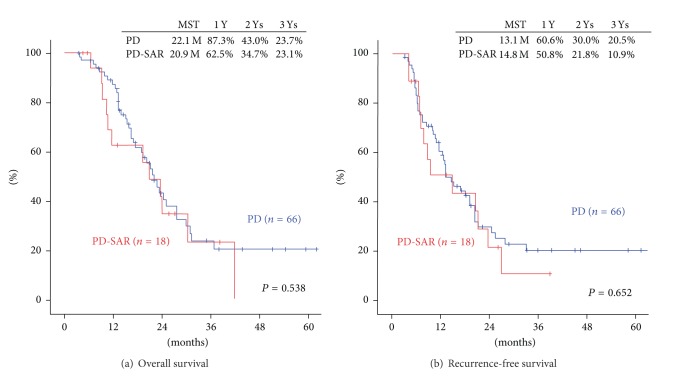
Comparisons of overall survival (OS) and recurrence-free survival (RFS) rates after pancreatectomy between PD and PD-SAR. (a) Overall survival. There were no significant differences in survival rates of two groups (*P* = 0.538). (b) Recurrence-free survival. There were no significant differences in survival rates of two groups (*P* = 0.652). MST: median survival time.

Median survival time (MST) and overall survival rates (OS) were almost similar between PD and PD-SAR: MST: 22.1 months versus 20.9 months and 3-year OS: 23.7% versus 23.1% (*P* = 0.538). Recurrence-free MST and recurrence-free rates (RFS) were also similar between PD and PD-SAR: MST: 13.1 months versus 14.8 months and 3-year RFS: 20.5% versus 10.9% (*P* = 0.652) (Figure 4).

### 3.5. Sites of Tumor Recurrence

Recurrence after operation occurred in 44 patients (66.7%) in PD and in 11 (68.8%) in PD-SAR, showing no significant difference. Although there were no significant differences in distant metastases between the two groups, the rate of local recurrence in the remnant pancreas was significantly higher in PD-SAR than in PD: 3/18 (18.8%) versus 2/66 (3.0%) (*P* = 0.030). The rate of recurrence in the remnant pancreas alone showed no significant difference: PD-SAR: 1/18 (6.3%) versus 0/66 (0%) (*P* = 0.483) (Table 4).

### 3.6. RPV and Type of DM Treatment

**Figure 5:**
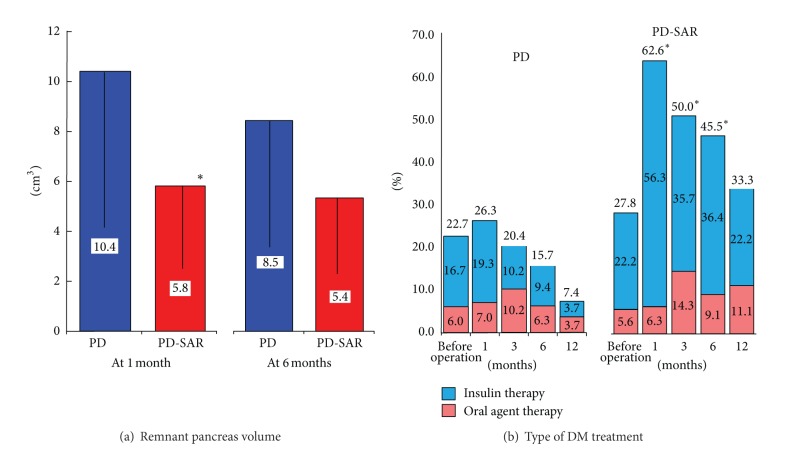
Comparisons of remnant pancreas volume (a) and type of postoperative DM treatment (b) between PD and PD-SAR. *P < 0.05 versus PD.

In PD and PD-SAR, the parenchyma of the remnant pancreas could be clearly enhanced in all patients. The RPV was significantly smaller in PD-SAR than in PD at 1 month after operation (5.8 ± 3.8 cm^3^ versus 10.4 ± 6.0 cm^3^, *P* = 0.029) but showed no significant difference at 6 months (5.4 ± 3.7 cm^3^ versus 8.5 ± 5.9 cm^3^, *P* = 0.199) (Figure 5(a)).

The percentage of patients who preoperatively required DM treatment was very similar between PD-SAR and PD: 27.8% (5/18) versus 22.7% (15/66). Postoperatively, however, the percentage became significantly higher in PD-SAR than in PD except for that of 12 months: 62.6% (10/16) versus 26.3% (15/57) at 1 month (*P* = 0.012), 50.0% (7/14) versus 20.4% (10/49) at 3 months (*P* = 0.027), 45.5% (5/11) versus 15.7% (5/32) at 6 months (*P* = 0.043), and 33.3% (3/9) versus 7.4% (2/27) at 12 months (*P* = 0.137). Additionally, the percentage of patients who postoperatively required insulin therapy was significantly higher in PD-SAR than in PD except for that of 12 months (*P* = 0.082) (Figure 5(b)).

### 3.7. Prediction of Postoperative Pancreatic Functions Using Several Markers

**Figure 6:**
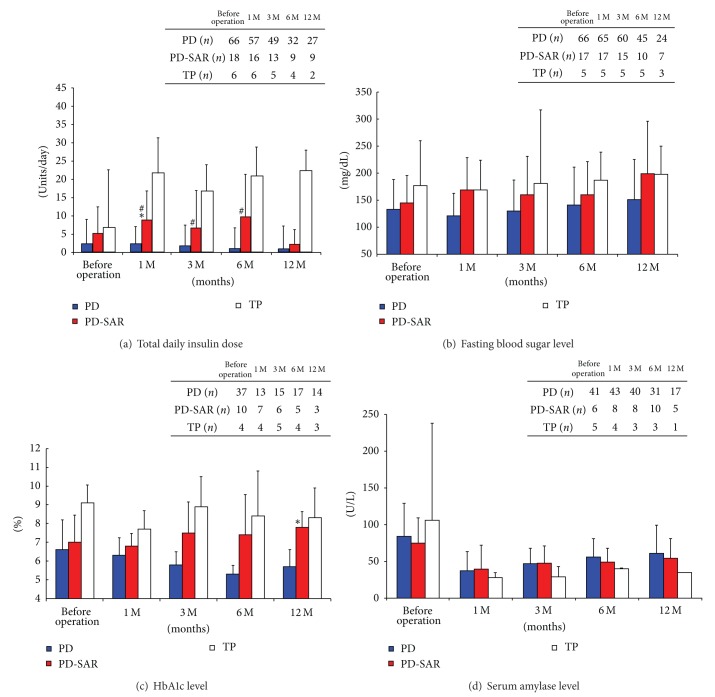
Markers for prediction of pancreatic functions before and after PD, PD-SAR, and TP. *P < 0.05 versus PD. ^#^ *P* < 0.05 versus TP.

**Figure 7:**
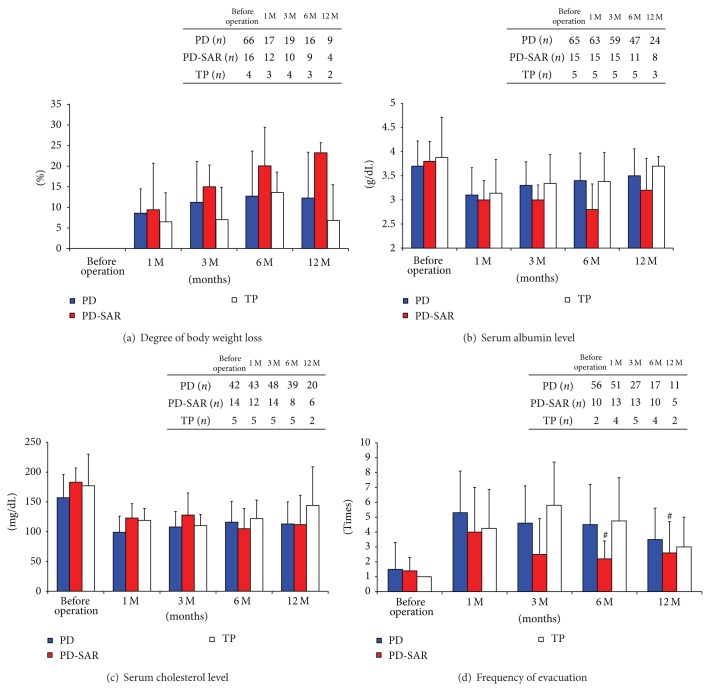
Nutritional markers for prediction of pancreatic functions and frequency of evacuation before and after PD, PD-SAR, and TP. *P < 0.05 versus PD. ^#^ *P* < 0.05 versus TP.

Total daily insulin dose (units) was significantly higher in PD-SAR than in PD at 1 month: 11.1 ± 13.1 versus 2.7 ± 6.7 (*P* = 0.026), while showing no significant differences between the two groups at 3, 6, and 12 months. As compared to TP, however, the dose in PD-SAR was significantly lower at 1, 3, and 6 months: 11.1 ± 13.1 versus 20.3 ± 5.4 (*P* = 0.024), 7.6 ± 13.3 versus 17.3 ± 3.1 (*P* = 0.025), and 10.1 ± 16.3 versus 26.3 ± 10.4 (*P* = 0.021) (Figure 6(a)). Fasting blood sugar, HbA1c, and serum amylase levels did not differ significantly among the three groups except for HbA1c levels at 12 months, showing significantly higher levels in PD-SAR than in PD: 7.8 ± 0.7% versus 5.6 ± 0.8% (*P* < 0.001) (Figures 6(b), 6(c), and 6(d)). No patients with PD and PD-SAR had experienced hypoglycemic attacks after discharge, while all patients with TP had experienced hypoglycemic attack after discharge. Degree of body weight loss and serum albumin and cholesterol levels did not differ significantly among the three groups (Figures 7(a), 7(b), and 7(c)). The degree of body weight loss seemed to be milder after TP than after PD-SAR, because in 6 patients in TP group including 2 with intraductal papillary mucinous adenocarcinoma body weight loss was minimal. Frequency of evacuation did not differ between PD-SAR and PD before and after operation, while it was significantly less in PD-SAR than in TP at 3 and 6 months: 2.5 ± 2.3 versus 5.8 ± 2.9 (*P* = 0.024) and 2.2 ± 1.2 versus 4.8 ± 3.0 (*P* = 0.011) (Figure 7(d)).

### 3.8. Glucagon Stimulation Test (GST)

**Figure 8:**
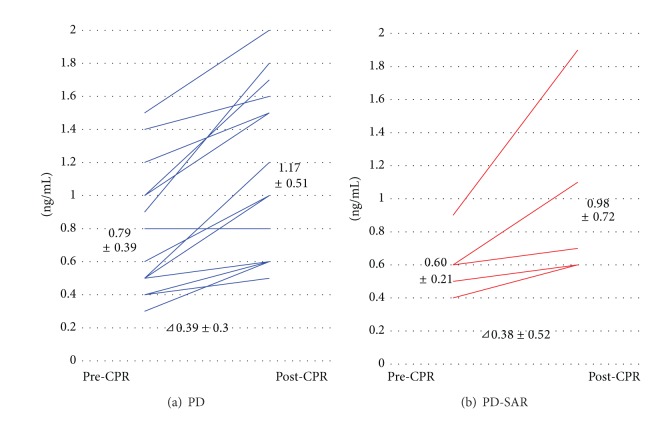
Glucagon stimulating test. CPR: serum levels of C-peptide immunoreactivity.

GST could be performed in 14 patients with PD and 5 with PD-SAR at 1 to 4 months after operation (median: 85 days). As a result, pre- and post-CPR levels (ng/dL) did not significantly differ between PD and PD-SAR: 0.79 ± 0.39 versus 0.60 ± 0.21 (*P* = 0.381) and 1.17 ± 0.51 versus 0.98 ± 0.72 (*P* = 0.692). Additionally, *⊿*CPR (ng/dL) showed no significant difference between the two groups: 0.39 ± 0.26 versus 0.38 ± 0.52 (*P* = 0.968) (Figure 8).

## 4. Discussion

For justification of PD-SAR procedure, sustained blood supply to the remnant pancreas is mandatory. Postoperative MDCT clearly demonstrated enhancement of the remnant pancreas at 1 and 6 months in all patients examined. Although we fortunately had not experienced any postoperative complications regarding lack of blood supply of the pancreatic parenchyma, it would be much better if the method to enhance blood supply of the remnant pancreas can be performed preoperatively. Hirano et al. [26] reported the usefulness of preoperative coil embolization of the common hepatic artery to enlarge the collateral pathways and prevent ischemia-related complications in patients who underwent distal pancreatectomy with en bloc celiac axis resection. Therefore, it might be also useful for PD-SAR patients to undergo preoperative coil embolization of the root of splenic artery for enhancing blood supply of the remnant pancreas. Furthermore, our present method to confirm blood supply of the remnant pancreas and spleen by macroscopic findings is unreliable and not objective, and therefore much more secure methods such as color Doppler ultrasound and indocyanine green fluorescence angiography [27] should be introduced in the future. Secondary point is, whether or not enough, surgical margin can be obtained by PD-SAR. As a result, there were no patients with pancreatic cut margin positive in both PD and PD-SAR and the sites of R1 were unexceptionally dissected margins around SMA and/or HA and/or CeA in both groups, although almost 90% of the patients in both groups had preoperative CRT. Additionally, surgical outcomes such as degree of postoperative complications and DHS did not differ between the two groups.

As of prognosis after PD-SAR, both OS and RFS were very similar to that after PD, despite the fact that the tumor size and the percentages of UICC-T4 and involvement of HA, CeA, and SA determined before treatment were significantly higher in PD-SAR. In contrast, there became no significant differences in pathological findings of the resected specimen including tumor size, T classification, lymph node metastasis, and degrees of lymphatic, venous, and intrapancreatic nerve invasions between the two groups. This was considered because preoperative CRT was effective to destruct tumor cells as shown in histological effect of CRT: the incidence of grade IIb or more (tumor destruction more than 50%) was higher in PD (45.8%) than in PD-SAR (18.8%) and incidence of grade IIa or more (tumor destruction more than 10%) was similar to each other (PD: 83.1% versus PD-SAR: 81.3%). In the present study, it was considered that preoperative CRT might enhance prognosis after PD-SAR. As shown in Table 3, the incidence of pathological T4, which means involvement of SMA and/or CeA, was 15.2% (10/66) in PD and 16.7% (3/18) in PD-SAR, which were markedly lower than 39.3% in PD and 66.7% in PD-SAR determined by MDCT before treatment (Table 1). Pathological diagnosis of arterial involvement of SMA and/or CeA was determined by presence of nerve plexus involvement in the dissected margin of SMA and/or CeA because combined resection of SMA and/or CeA was not performed. On the other hand, arterial involvement of SMA and/or CeA determined by MDCT depended on imaging findings such as tumor abutment and/or encasement. Mochizuki et al. [28] examined MDCT findings of extrapancreatic nerve plexus invasion around SMA by “point-by-point” correlation with en bloc pathological specimens to assess their diagnostic accuracy in 37 patients with PDAC including 16 with combined resection of SMA. As a result, diagnostic accuracy of nerve plexus invasion around SMA reached 94.6%. In the present study, histological effect of CRT showed that the incidence of grade IIb or more was 45.8% in PD and 18.8% in PD-SAR. Taken these facts together, it was likely assumed that preoperative CRT reduced incidence of pathological arterial involvement.

When we compared mode of tumor recurrence between PD and PD-SAR, the rate of local recurrence in the remnant pancreas was significantly higher in PD-SAR (18.8%) than in PD (3.0%). Among 3 patients with local recurrence of the remnant pancreas, 2 had distant metastasis simultaneously and died at 5 and 8 months after PD-SAR, respectively, and 1 had local recurrence of the remnant pancreas alone and died at 18 months. These results suggested that local recurrence of the remnant pancreas after PD-SAR might not affect long-term survival, although further study to accumulate number of cases is required. According to the study of Schmidt et al. [29], on the oncologic benefit of conversion of PD to TP to achieve an R0 resection in PDAC patients with an isolated positive cut margin of the pancreas, 28 patients underwent PD with an isolated positive cut margin without additional resection, while 33 patients had conversion to TP for isolated cut margin involvement to achieve R0 resection. As a result, patients undergoing TP versus PD had a greater MST (18 versus 10 months, *P* = 0.04). Therefore, they concluded that conversion of PD to TP to achieve an R0 resection was associated with a survival benefit. In PD-SAR, however, we made the pancreatic cut line as distal as possible to achieve a negative cut margin, and fortunately all patients except for 2 could obtain negative pancreatic margin and avoid TP. As a result, both OS and RFS were very similar to that after PD.

To the best of our knowledge, there have been no previous reports to examine the postoperative changes of the RPV after PD, although a few studies [30, 31] measured it at one point of time. We examined RPV at 1 and 6 months after pancreatectomy. As a result, RPV was significantly smaller in PD-SAR than in PD at 1 month but showed no significant difference at 6 months. Comparing RPV between 1 and 6 months after PD, it decreased from 10.4 ± 6.0 to 8.5 ± 5.9 (*P* = 0.079), while after PD-SAR it did not change from 5.8 ± 3.8 to 5.4 ± 3.7. These results demonstrated that small remnant pancreas after PD-SAR kept the volume almost unchanged until 6 months, indicating the significance of PD-SAR.

Because RPV after PD-SAR becomes almost half of PD, it was predicted that insulin therapy becomes a big problem after PD-SAR. However, total daily insulin dose was significantly higher in PD-SAR than in PD at 1 month alone, while showing no significant differences between the two groups thereafter. As compared to TP, however, the dose in PD-SAR was significantly lower, and no patients after PD-SAR had experienced hypoglycemic attacks, while all patients after TP had experienced it. Recently, Barbier et al. [3] reported short- and long-term outcomes of 56 patients with TP. In their study, 40% of the patients had a loss of consciousness owing to hypoglycemia and all patients had experienced a median of 10 hypoglycemic episodes per month. Furthermore, 5 deaths were related to TP (two postoperative deaths, one hypoglycemia, one ketoacidosis, and one anastomotic ulcer). They conclude that endocrine and exocrine insufficiency after TP impacts on the long-term QOL. Ourpresent studies on the prediction of postoperative pancreatic functions using several makers revealed no significant differences between PD and PD-SAR except for HbA1c levels at 12 months, and these results suggested that PD-SAR maintained long-term QOL. Finally, we performed GST in selected patients with PD and PD-SAR to confirm insulin secretion ability from the remnant pancreas. Pre- and post-CPR levels and *⊿*CPR did not significantly differ between the two groups, revealing enough insulin secretion ability from the remnant pancreas after PD-SAR. In the present study, we did not examine the measurement of future RPV before surgery. It is considered that preoperative measurement of future RPV is useful to predict postoperative pancreatic functions and development of fatty liver (nonalcoholic fatty liver disease: NAFLD) after PD, as we reported that the RPV less than 10 mL at 1 month is a strong predictor of NAFLD after PD [32].

Recently, it has been recognized that TP with islet cell autotransplantation is an effective surgery for end stage of chronic pancreatitis [33]. However, the possibility of infusion of occult carcinoma cells in the islet preparation restricts the use of this procedure to treat PDAC. In 2001, Liu et al. [34] reported the first successful case of islet cell autotransplantation combined with TP for treatment of PDAC. At 1-year follow-up, HbA1c was 6.2% although the patient remained insulin dependent (18 U/d). The pre- and post-CPR levels (ng/dL) in GST were 0.66 and 0.84, respectively, which were comparable to our data after PD-SAR: 0.60 ± 0.21 and 0.98 ± 0.72. Although islet cell autotransplantation combined with TP for PDAC seems to be feasible, our PD-SAR to avoid TP has broad utility to treat PDAC in terms of oncological safety, simplicity, and low cost.

In conclusion, PD-SAR with preoperative CRT seems to be promising surgical strategy for PDAC of head and/or body with invasion of the splenic artery, in regard to the balance between operative radicality and postoperative QOL.

## Figures and Tables

**Table 1:** Comparison of preoperative characteristics between PD and PD-SAR.

Variable	PD (*n* = 66)	PD-SAR (*n* = 18)	*P* value
Gender			
Male	43	6	**0.029**
Female	23	12	
Age (years)	66.5 ± 9.6	67.6 ± 9.2	0.718
Tumor size before treatment (mm)	30.8 ± 8.7	37.8 ± 10.9	**0.030**
UICC-T3	40 (61.7%)	6 (33.3%)	**0.060**
UICC-T4	26 (39.3%)	12 (66.7%)	
Resectability			0.124
R	6	1	
BR	49	10	
UR	11	7	
Cancer involvement of major vessels			
SMV/PV	59 (89.4%)	16 (88.9%)	0.713
SMA	20 (30.3%)	5 (27.8%)	0.934
HA	8 (12.1%)	8 (44.4%)	**0.006**
CeA	6 (9.0%)	6 (33.3%)	**0.026**
SA	0 (0%)	18 (100%)	**<0.001**
Ao/IVC	2 (3.0%)	1 (5.6%)	0.838
Treatment before surgery			
G-CRT	35 (53.0%)	11 (61.1%)	0.917
GS-CRT	24 (36.4%)	5 (27.8%)	
CTA	3	1	
Non	4	1	
CA19-9 levels (U/mL)			
Before preoperative treatment	620.7 ± 1710.5	681.9 ± 1803.3	0.878
After preoperative treatment	87.7 ± 77.2	155.6 ± 335.0	0.216

UICC: International Union for Cancer Control; R: resectable; BR: borderline resectable; UR: unresectable; SMV: superior mesenteric vein; PV: portal vein; SMA: superior mesenteric artery; HA: hepatic artery; CeA: celiac artery; SA: splenic artery; Ao: aorta; IVC: inferior vena cava; G-CRT: gemcitabine-based chemoradiotherapy; GS-CRT: gemcitabine plus S1-based chemoradiotherapy; CTA: chemotherapy alone; Non: no treatment before surgery.

**Table 2:** Comparison of surgical outcomes between PD and PD-SAR.

	PD (*n* = 66)	PD-SAR (*n* = 18)	*P* value
Blood loss (g)	1967 ± 1874	1605 ± 1215	0.340
Operation time (min)	587 ± 118	607 ± 127	0.429
Combined resection			
SMV/PV	58 (87.9%)	18 (100%)	0.271
Colon	7 (10.6%)	0	0.336
Total gastrectomy	1 (1.5%)	2 (11.1%)	0.219
HA	3 (4.5%)	1 (5.6%)	0.656
SA	0 (0%)	18 (100%)	**<0.001**
Type of P-J anastomosis			
PWST	65 (98.5%)	12 (61.1%)	**<0.001**
Dunking	1 (1.5%)	6 (38.9%)	
Blood transfusion (mL)	400 ± 420	320 ± 406	0.660
Postoperative complication			
C-D grade ≥ III	13 (19.7%)	3 (16.7%)	0.790
DHS (days)	40.2 ± 17.9	38.2 ± 13.5	0.980

P-J: pancreaticojejunostomy; PWST: pair-watch suturing technique (16); C-D: Clavien-Dindo classification (18); DHS: duration of hospital stay.

**Table 3:** Comparison of pathological findings of resected specimen between PD and PD-SAR.

	PD (*n* = 66)	PD-SAR (*n* = 18)	*P* value
Tumor size (mm)	26.3 ± 10.2	31.6 ± 10.9	0.098
UICC-T1	8	1	0.869
UICC-T2	12	3	
UICC-T3	36	11	
UICC-T4	10	3	
UICC-stage			
IA/IB/IIA/IIB/III/IV	5/4/22/24/10/1/0	1/2/7/5/2/0/1	0.529
JPS-stage			
I/II/III/IVa/IVb	5/7/30/24/0	1/2/8/6/1	0.435
Histological type			
Well	30	11	0.108
Moderate	29	4	
Poor	7	2	
Other	0	1	
Lymph node metastasis			
Positive	27	4	0.117
Negative	39	14	
Degree of lymphatic invasion*			
ly0	17	5	0.754
1–3	44	10	
Degree of venous invasion*			
v0	42	9	0.358
1–3	19	6	
Degree of intrapancreatic nerve invasion*			
ne0	17	4	1.000
1–3	44	11	
Histological effect of CRT (Evans' criteria)			
I	10	3	0.083
IIa	22	10	
IIb	21	3	
III, IV	6	0	
Status of surgical margin			
R0	56	14	0.150
R1	9	2	
R2	1	2	

UICC: International Union for Cancer Control; JPS: Japan Pancreatic Society; ly: degree of lymphatic invasion; v: degree of venous invasion; ne: degree of intrapancreatic nerve invasion; R0: negative surgical margin; R1: positive microscopic margin; R2: positive gross margin. *Excluding 8 cases in which histological assessment could not be determined.

**Table 4:** Comparison of tumor recurrent sites between PD and PD-SAR.

	PD (*n* = 66)	PD-SAR (*n* = 18)	*P* value
Recurrence	44 (66.7%)	11 (68.8%)	0.873
Local			
Remnant pancreas*	2 (3.0%)	3 (18.8%)	**0.109**
Remnant pancreas alone	0 (0%)	1 (6.3%)	0.483
Others	4 (6.1%)	2 (12.5%)	0.825
Metastasis			
Liver	15 (22.7%)	3 (18.6%)	0.817
Lung	10 (15.2%)	2 (12.5%)	0.957
Lymph node	2 (3.0%)	1 (6.3%)	0.838
Dissemination	9 (13.6%)	3 (18.6%)	0.957

*Recurrence of remnant pancreas associated with metastasis of other organs.
